# Reducing violent discipline by teachers using Interaction Competencies with Children for Teachers (ICC-T): study protocol for a matched cluster randomized controlled trial in Tanzanian public primary schools

**DOI:** 10.1186/s13063-019-3828-z

**Published:** 2020-01-02

**Authors:** Faustine Bwire Masath, Katharin Hermenau, Mabula Nkuba, Tobias Hecker

**Affiliations:** 1 Department of Psychology, Bielefeld University, 33501 Bielefeld, Germany; 2 Department of Educational Psychology and Curriculum Studies, Dar es Salaam University College of Education, 2329 Dar es salaam, Tanzania; 3 vivo international, Postbox 5108, 78340 Constance, Germany

**Keywords:** Violent discipline, Intervention, Violence prevention, ICC-T, Primary schools, Teachers, Students

## Abstract

**Background:**

Despite the existing national and international plans of action to end violent discipline strategies used by teachers in schools, they still prevail in Tanzanian schools. This underlines the need to implement school-based interventions that aim at reducing violent discipline by teachers. In this study, we will evaluate the feasibility and effectiveness of the preventative intervention *Interaction Competencies with Children – for Teachers (ICC-T)* in Tanzanian primary schools. Following its success in secondary schools, we hypothesize that the intervention will reduce teachers’ positive attitudes towards and their use of violent discipline at school.

**Methods/design:**

The study will be conducted in six randomly selected regions in Tanzania. We have already randomly selected two schools in each region (12 in total) that fulfill our inclusion criteria. From each region, one school will be randomly assigned to the intervention and the other to the monitoring group (no intervention). Eighty students between the ages of 9 to 12 years (*N* = 960) and 20 teachers from each school (*N* = 240) will be included in the trial. We will collect data directly before the intervention (t1) and 6 months after the intervention (t2) both at intervention and monitoring schools. Using guided questionnaire assessments, we will measure violence by teachers using students’ reports on their exposure to and teachers’ reports on their use of violence using the Conflict Tactics Scale. Furthermore, we will assess teachers’ positive attitudes towards violent discipline using a modified version of the Conflict Tactic Scale. The feasibility of the intervention will be evaluated using purpose-built measures assessing the demand, applicability, acceptability, and integration of core elements into daily work in the participating schools.

**Discussion:**

The proposed study will allow us to test the feasibility and effectiveness of an intervention aiming to reduce positive attitudes towards and the use of violent discipline by teachers in school settings. With the reduction of violent discipline by teachers, this study contributes to national and international efforts towards ending violence against children as well as the attainment of the United Nations’ Sustainable Development Goals that also aim to prevent all types of maltreatment of children.

**Trial registration:**

ClinicalTrials.gov, NCT03893851. Registered on 28 March 2019.

## Background

Violent discipline by teachers at school entails the use of different forms of physical and emotional acts that violate children’s dignity and inflict pain with the intention of correcting a child’s misconduct [1]. Violent discipline takes diverse forms, including the use of hands or objects, such as a cane, whip, or stick, by teachers and school staff [2]. Despite the existence of different national and international intervention strategies to end violent discipline by teachers, the prevalence of the use of violent discipline by teachers is still reported globally [3–5]. In many countries in Sub-Saharan Africa, including, for example, Tanzania, violent discipline by teachers is still legal and considered by teachers as being a necessary and effective disciplinary tool to control student behavior [6].

### Global perspective on violence by teachers at school

Previous research has indicated that violence against children in schools is a critical global problem [7]. Straus [8] reported that millions of children worldwide experience violence at school. For example, the prevalence of violence by teachers ranged from 34% to 93% in India, from 7% to 51% in Peru, from 1% to 50% in Vietnam, from 12% to 76% in Ethiopia, and was reported to be 58% in Jamaica and 56% in Yemen [9–11]. These findings stand in direct contrast to the aims of the United Nations’ Convention on the Rights of the Child [12], which outlines the “protection of the physical and mental integrity of all children”, as well as of the United Nation’s Sustainable Development Goal No. 16.2 [13] which aims to “end all forms of violence against children”. Nevertheless, Zolotor and Puzia [14] and the Global Initiative to End All Corporal Punishment against Children [3] reported that corporal punishment and other forms of violence against children in a school setting are legally prohibited in 128 countries while 69 countries still legally accept such violent actions as a means of controlling students’ misconduct at schools, leaving slightly more than two billion children worldwide without full legal protection.

Reports of violence against children in Africa differ significantly from one country to another [15]. In Uganda, for instance, the use of harsh discipline is prevalent among teachers in schools despite the prohibition by law [16]. Exposure to teachers’ violent discipline was reported by 86% of girls in Kenya, 82% in Ghana, and 66% in Mozambique [17]. In Uganda, school children are reportedly at high risk of all forms of violence, particularly physical and emotional violence by school staff [18]. In Tanzania, UNICEF [19] reported that one out of two children reported having experienced physical violence at the hands of teachers. For example, children were frequently beaten and even burnt by their teachers [20]. A recent study of a national representative sample of Tanzanian secondary school students reported that almost every student experienced physical and/or emotional violence by teachers. This very high prevalence was reported both by students and teachers [5, 21].

### Factors influencing the use of violent discipline by teachers

The use of violent discipline at school in many of these countries can be attributed to a number of factors, including large classes and inadequate training in how to interact non-violently with students [16]. In fact, the teaching and learning environment in East Africa is a harsh one currently, with stressed and burdened teachers, poor teacher–student ratios, and limited classroom infrastructure, all of which seem to increase the likelihood of teachers employing violent discipline strategies [1, 5, 6, 22]. Hecker et al. [5] reported that teachers’ stress as well as lack of alternative disciplining strategies among teachers intensified the use of violent discipline among teachers. Furthermore, Meyer and colleagues [23] reported that a lack of a positive school environment and children’s exposure to community violence—which itself was linked to adverse mental health outcomes—were associated with the use of violent discipline by teachers at school in low income countries, such as Tanzania. Furthermore, teachers’ beliefs, poor working conditions, and problems in teacher–student interactions increased teachers’ use of violent discipline [6]. Moreover, Semali and Vumilia [24] put forth that teachers considered the use of violent discipline as an accepted norm. Teachers are reported as believing that violent discipline is a useful method for creating an orderly learning environment, developing good conduct, maintaining safe school behaviors, enforcing discipline, and improving academic performance. Teachers justify the use of violence in the context of child discipline procedures and as a way of exercising power, compliance, and behavioral control [25]. Cheruvalath and Tripathi [26] argue that a lack of proper training on how to manage students and a poor understanding of the consequences of violence for school children may significantly increase the use of violent discipline by teachers. In addition, violence against children in Tanzanian school settings is still high due to the legalization of violent discipline [1, 5] as well as the social acceptance of violent discipline in the community at large and by teachers specifically [6, 27].

### Effects of violence on child development

The learning environment should be free of fear and violence to ensure children’s healthy physical, mental, social, emotional, and cognitive development, which together contribute to the academic performance of students [28]. The socio-ecological theory explains children’s development as a process embedded within multiple contexts, which include families, schools, and communities [29]. Therefore, it is important to examine school contexts to better understand the effects of violence by teachers. In schools, the high prevalence of violence affects students in all grades [29, 30]. For example, school violence is associated with lower academic performance [31–34], impacts children’s cognitive development [35–37], and is related to mental health problems [31, 34, 38–40]. Furthermore, children experiencing violence and maltreatment in schools displayed significantly higher levels of psychological problems, including depression, loneliness, anxiety, reinforcement of negative self-evaluations, and fear or avoidance of social interactions and, as a result, they increasingly became targets of bullying by peers [40–42].

### Global intervention strategies to end violence against children

Globally, different initiatives have been carried out in an effort to protect children from violence. The United Nations General Assembly [12] set the agenda to end all forms of violence against children almost 30 years ago. Article 19 of the United Nations Convention on the Rights of the Child calls for the protection of children from all forms of physical or emotional violence, injury, or abuse by any person in any setting. Article 28 requires parties to take all appropriate measures in ensuring that the behavior of children in schools is managed in a manner consistent with the child’s human dignity [12]. Moreover, the Global Initiative to End All Corporal Punishment against Children [43] aims to eliminate violence against children in all settings and goes hand in hand with UN Sustainable Development goal No.16.2.

Besides initiatives that aim at contributing to legal and political changes, very few school-based intervention strategies that aim to reduce the use of violent discipline in school settings have been implemented and even fewer have been scientifically evaluated in Sub-Saharan Africa. In Uganda, for example, the Good Schools Toolkit, which includes different activities for staff, students, and administration, focused on improving the school compound and creating a better learning environment, respect and understanding power relationships, improving teaching techniques, creating accountability, and learning non-violent methods of discipline. The intervention has been used in primary schools and its effect on overall levels of ‘any’ form of violence (physical, emotional, sexual combined) from school staff and/or peers has been reported [16, 44, 45]. Another example is the intervention approach Stop Violence Against Girls in Schools that was carried out by Action Aid in Ghana, Kenya, and Mozambique. The intervention program showed significant results: the use of caning by teachers dropped by 23% in Mozambique while girls enrolment increased by 14%, 17%, and 10% in Ghana, Kenya, and Mozambique, respectively [46].

### Intervention to end violence by teachers against children in Tanzania

In Tanzania, severe forms of violence against children in school settings are generally prohibited by the Law of the Child Act [47], which states: “A person shall not subject a child to torture, or other cruel, inhuman punishment or degrading treatment including any cultural practice which dehumanizes or is injurious to the physical and mental well-being of a child ….”*.* Moreover, the Corporal Punishment Guideline [48, 49] set limits to the number of strokes from six to four and gives mandate to the Head of School to administer the punishment. Additionally, the guideline directs the Head of School to document details of the student being punished to include, among other details, names and the misbehavior committed. However, in reality we find differences in implementation of the legal guideline as teachers administer corporal punishment without compliance to the law and the guideline [49].

Apart from the legal intervention strategies, the school-based preventative intervention approach *Interaction Competencies with Children – for Teachers* (*ICC-T*) has been evaluated in a cluster randomized controlled trial (CRCT) in secondary schools and in a pilot study with one selected primary school in Tanzania [1, 6]. *ICC-T* is based on attachment, behavioral, and social learning theories. The key principles are the intervention’s feasibility in low resource contexts, a participatory approach, and practical orientation. *ICC-T* aims to enable teachers to use non-violent disciplinary measures and to strengthen their competencies in non-violent interactions by introducing essential interaction competencies with children into the daily work of teachers. The pilot study demonstrated the feasibility of *ICC-T* at the primary school level [6] and the CRCT provided initial evidence of the effectiveness of the preventative intervention at the secondary school level: The use of violent discipline by teachers (self-reported and reported by students) was reduced in the intervention schools compared to control schools. Furthermore, teachers in the intervention schools reported less positive attitudes towards the use of violent discipline in intervention schools at follow-up compared to control schools [1]. Participating teachers in intervention schools reported a high acceptance of the intervention and a good integration of *ICC-T *content into their daily work.

### Study objective

Based on the lack of evidence for school-based interventions — particularly at the primary school level — that effectively reduce violence utilized by teachers, we advocate for conducting controlled intervention studies to reduce the use of violent discipline by teachers in primary schools. Reducing violence against children in education settings requires intervention strategies that also target a change in common societal beliefs and attitudes that the community holds towards violent discipline. Due to the legalization of violent discipline in Tanzanian schools under the National Corporal Punishment Regulations pursuant to article 60 of the National Education Act [48, 49], positive attitudes are still associated with the use of violent disciplinary methods. Furthermore, teachers lack non-violent action alternatives. Therefore, there is a pressing need to implement preventative intervention approaches and to conduct controlled studies aiming to reduce the use of violent discipline by teachers in primary schools in Tanzania. Embarking on the success of *ICC-T* in secondary schools in Tanzania [1], in this study we therefore aim to test the feasibility and effectiveness of *ICC-T* in primary schools. We therefore hypothesized that the implementation of *ICC-T* will reduce both positive attitudes towards and the use of violent discipline by teachers in primary schools in Tanzania.

## Methods/design

### Design of the study

**Fig. 1:**
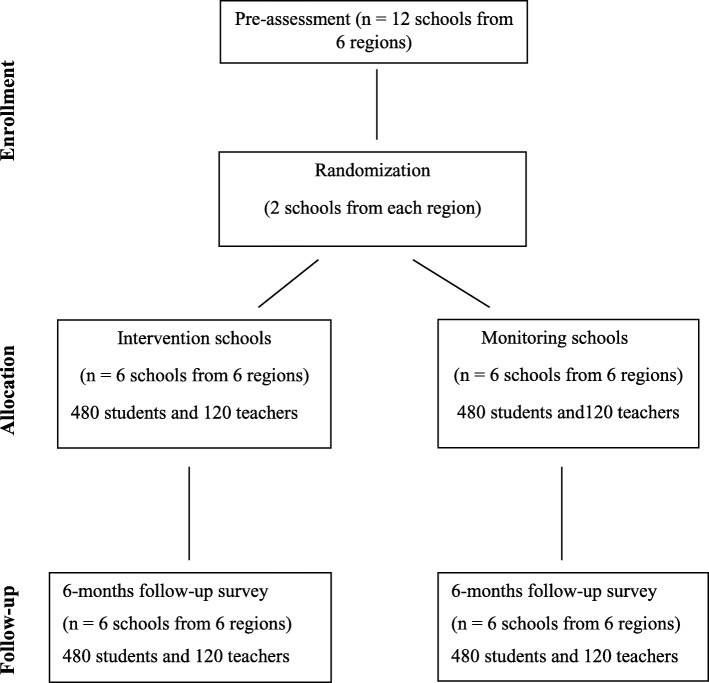
Flow chart of the study design

**Fig. 2:**
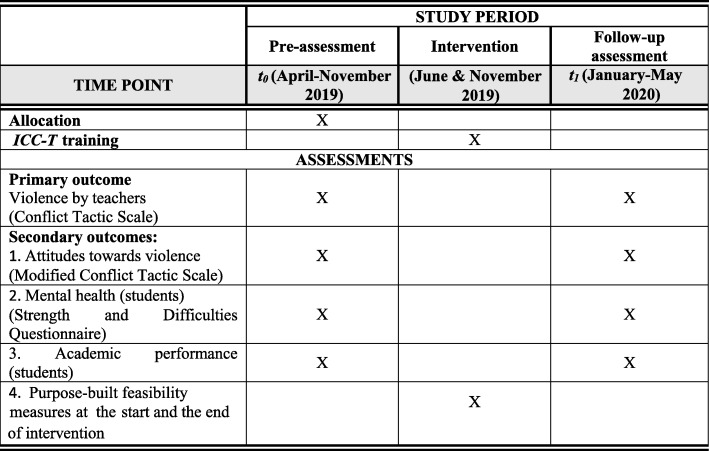
Participant time line chart

Using a two-arm matched cluster randomized controlled trial, the study will include 12 schools from six regions in Tanzania. From each region one school will be randomly allocated to the intervention school condition (from which all teachers will be trained in an ICC-T intervention workshop) and the other to the monitoring school condition (which receive no intervention). The study will have two data collection phases: pre-assessment directly before the intervention (t0) and follow-up assessment approximately 6 months after intervention (t1). See Figs. 1 and 2 and the SPIRIT 2013 Checklist: Recommended items to address in a clinical trial protocol and related documents (Additional file 1).

### Study setting

This study will be carried out in public primary schools in Tanzania. In total, five regions have been randomly selected to represent the five administrative zones: Central-Western Lake Zone, Eastern and Coast Zone, Lake Zone, Northern Zone, and Southern Highland Zone*.* Using http://www.random.org, the regions Tabora, Mtwara, Shinyanga, Tanga, and Njombe have been randomly selected, respectively, to represent the aforementioned zones. In addition, the Dar es Salaam region will be studied due to its unique population characteristics and its significant number of schools, teachers, and students at the primary school level [50]. The selection of the regions ensures representation of the country geographically, economically, socially, and politically. From each region, two districts were randomly selected: Igunga and Urambo from Tabora region; Mtwara and Newala from Mtwara region; Kishapu and Shinyanga from Shinyanga region; Korogwe and Tanga from Tanga region; Njombe and Wanging’ombe from Njombe region; and Kinondoni and Temeke from the Dar es Salaam region.

### Schools

One public primary school from each selected district will be randomly selected, resulting in a total of 12 schools. Only public primary schools with more than 40 students per enrolment year will be included in the study. The list of schools with the specified criteria was obtained from the National Examination Council of Tanzania [51]. The number of students who sat the primary school leaving exam in each of the respective district’s schools implied their enrolment rate. Schools that qualified for the criteria in each district will be listed in alphabetical order and a random selection of one school from each district will be executed using http://www.random.org.

### Participants

We will assess children and teachers at pre-assessment and at follow-up assessment. In addition, feasibility data of teachers participating in intervention trainings will be assessed at the beginning and the end of the *ICC-T* training workshops as well as at follow-up assessment. We aim to include students from the fifth and sixth years of formal schooling. Students’ ages will range between 9 and 12 years. This age group is selected because of its ability to comprehend the questionnaire items and its availability during follow-up assessment (students in the seventh year of formal schooling will shift to secondary school before the follow-up assessment can be completed). All teachers employed at the selected schools will be eligible to participate in the study (Fig. 1).

Within each of the selected schools, 40 pupils from of the fifth and 40 pupils from sixth year of formal schooling will be stratified by gender and then randomly selected. Thus, the target sample will be 960 pupils. Based on a study that used a similar design [1], we would expect moderate to large effects on pupils’ self-reported exposure to violence. An a priori power analysis (α = 0.05, power = 0.80, moderate effect size of ƒ = 0.25) indicated a required total sample size of at least *n* = 128 pupils to detect significant interaction effects. To adjust for the nested design of the study, we calculated the following design effect (DE): DE = 1 + (fixed cluster size considering drop-outs − 1) × intra-cluster correlation coefficient. Considering 80 pupils per school, a dropout rate of 20%, and an intra-cluster correlation coefficient of 0.05, the DE for the pupil sample is 4.15, which results in a required sample size of at least 532 pupils. All teachers at the selected schools will be included in the study sample. Our target sample will be at least 20 teachers per school, resulting in a total sample of at least 240 teachers. Based on the previous study, we would also expect moderate to large effects on teachers’ self-reported use of violence against students. The a priori power analysis (see above for details) indicated a required total sample size of at least *n* = 128 teachers to detect significant interaction effects. To adjust for the nested design of the study, we again calculated the DE. Considering a minimum of 20 teachers per school, a drop-out rate of 20% and intra-cluster correlation coefficient of 0.05, the DE for the teacher sample is 1.8, which results in a required sample size of at least 231 teachers.

### Procedures

Before data collection, the research team will train four Swahili speaking research assistants in data collection. They will be blind regarding the allocation of the schools. A standardized introduction and questionnaire administration procedures will be developed to ensure high objectivity and reliability during data assessment. All questionnaires will be administered in Swahili. Following established international guidelines [52], Swahili native speakers will translate all measures that are not available in Swahili from English to Swahili and then back to English in a blind written form. The back-translated measures will then be compared with the original measures to ensure correct translation and equivalence of the content. A pilot study at one primary school (not included in the trial) will be conducted to ensure the feasibility of the questionnaire administration.

Prior to data collection, a letter explaining the study aims and procedures will be sent together with an informed consent form to the parents to seek parental consent. To ensure common and clear understanding of the relevant details of the study, the research team will provide information to all selected students in a formal information session. Students who provide assent and whose parents have signed the informed consent form will be invited to fill out questionnaires in groups of two to four students on the school grounds under supervision of a research assistant. To ensure sufficient privacy, the students will be seated so that it will be impossible for anyone to see how the questions are being answered. The completion of questionnaires will take an average of 45 min at baseline and follow-up.

After being introduced to the project’s objectives, the teachers will be invited to participate in the study. Teachers willing to participate will be requested to sign an informed consent form and to fill out a questionnaire in their free time between lessons under the guidance of an assessor in a one-to-one setting. The completion of the questionnaires will take, on average, 30 min at baseline and at follow-up assessment.

### Intervention

In the intervention schools, the *ICC-T *intervention will be implemented for 5.5 days (8 h on a full day).* ICC-T* is based on attachment, behavioral, and social learning theories. *ICC-T* includes sessions on (a) teacher–child communication and interaction, (b) violence prevention, (c) effective non-violent discipline strategies, and (d) identifying and supporting burdened children (for more details see [1, 2, 6]). *ICC-T* intervention in the selected schools will be implemented by one main facilitator with the assistance of two assistant facilitators. All facilitators are trained Tanzanian psychologists and teachers. *ICC-T* materials for training as well as presentations and discussion during trainings will be in Swahili. Participation of the training will be free of charge. Food and beverage will be provided to participants as well as transport compensation of US$4 per day. Prior to the official participation in the training, teachers will be offered introductory letters informing them of the voluntary nature of their participation as well as their right to withdraw from the training at any point. Teachers who have agreed to participate will sign the training informed consent form. Confidentiality of information shared during the training will be ensured. No personal information will be shared with external sources.

### Control

At the monitoring schools no intervention will be implemented. The research team will also have close contact with the monitoring schools to ensure that teachers do not receive any training of the same nature during the study duration. Data assessment at monitoring schools will be conducted both at pre-assessment and follow-up phases.

### Outcome measures

Our study intends to test the effects of *ICC-T* on the use of violent discipline by teachers at school. This primary outcome measure will be assessed by students’ self-reported experiences of violence as well as teachers’ self-reported use of violence. Secondary outcome measures include teachers’ attitudes towards violent discipline, children’s mental health and well-being, as well as students’ school performance (provided by the school administration). Purpose-built measures adapted from previous studies [1, 6] will be used to assess the feasibility of ICC-T at the primary school level. We follow the guidelines for feasibility studies in assessing the demand, applicability, acceptability, and integration of *ICC-T* core elements in the teachers’ daily work [53].

#### Children

##### Experience of violence

At pre-assessment and at follow-up assessment, we will use the Conflict Tactic Scale (CTS) [54] to capture student’s experiences of violence by teachers. Students’ exposure to violence by teachers will be assessed using the physical violence and the emotional violence subscales of the CTS. Thirteen items assessing physical violence and five items measuring emotional violence subscales will be applied. The scale items are answered on a seven-point Likert scale ranging from 0 (this has never happened) to 6 (more than 20 times) and the sum score ranges from 0 to 78 for physical violence and from 0 to 30 for emotional violence. Subscale scores are derived by summing up all item scores. The CTS presented with acceptable internal consistency of 0.88 in a Ugandan student sample [22]. The CTS scale has been used in East African student samples and has demonstrated its utility in assessing students’ exposure to violence by teachers [1, 5, 22].

##### Mental health problems

We will use the Strength and Difficulties Questionnaire (SDQ) to assess children’s mental health problems at pre-assessment and at follow-up assessment. The items are assessed using a three-point Likert scale ranging from 0 (not true) to 2 (certainly true). Reversed items are recorded before the computation of the total scale score (sum of scores for all items excluding the five items of the prosocial behavior subscale) that ranges between 0 and 40, with a score above 20 representing the presence of mental health problems. The Cronbach alpha reliability of the total difficult score was 0.82 in the validation study [55]. The SDQ scale has been tested in the Tanzanian population and proved its usefulness in screening for mental health problems [34, 39, 40, 56].

##### Academic performance

In addition, we will assess students’ academic performance. The students’ scores in mathematics, Swahili, English, science, social science, and general studies in the mid-term exam will be provided by the school administration.

#### Teachers

##### Use of violent discipline

We will use a modified version of the CTS which was initially developed to assess parents’ reports on their use of violence to assess teachers’ use of physical and emotional violence in schools (see above for more details on subscales and answer categories). The scale has been implemented in similar studies in East Africa [1, 2]. The CTS presented with acceptable internal consistency of 0.76 in a Ugandan teacher sample [22]. The CTS scale has proved its usefulness in assessing teachers’ self-reported use of violence in the classroom [5, 22].

##### Attitude towards discipline

We will use an adaptation of the CTS to assess teacher’s positive attitude towards the use of violent discipline [1]. The items are the same as described before but this time answered on a four-point Likert scale ranging from 0 (never OK) to 3 (always or almost always OK). The scores for each subscale are then summed up to one score for physical assault and one for emotional violence. In a study with Ugandan teachers the Cronbach alpha coefficient was 0.80 for the total score [22]. The modified CTS scale has been used in East African teacher samples and it proved its usefulness in assessing teachers’ self-reported attitudes towards violence in the classroom [1, 22].

##### Purpose-built measures for ICC-T training evaluation

We will adopt the purpose-built measures as used in previous studies conducted by our team [1, 2, 6]. We will follow the guidelines for feasibility studies by Bowen et al. [53] in assessing the demand, applicability, acceptability, and integration of *ICC-T* training techniques into teachers’ daily work. We will assess participant’s expectations regarding the workshop and its relevance in their daily work before the intervention, directly after the intervention, and at the follow-up stage. Additionally, acceptability of the training (i.e., satisfaction with the training and evaluation of new knowledge acquired) will be assessed after the intervention and at the follow-up stage. Lastly, teachers’ incorporation of the *ICC-T *core elements into their daily school work will be assessed after the intervention and at the follow-up stage.

### Measures against bias

The stratified random sampling approach will minimize recruitment bias. Our careful selection of the assessment instruments will minimize bias based on the use of unvalidated outcome measures. As the allocation will be executed at the cluster level and by the core research team, those conducting data collection will be blind to the treatment conditions of the schools. Though the intervention participants will not be blind in regard to whether they belong to the intervention or the monitoring group, violence by teachers will also be assessed by assessing students’ self-reported exposure to violence. Analysis will be carried out based on the groups as randomized (‘intention to treat’) to avoid incomplete accounting of participants and outcome events. The trial has been registered in a trial registry for clinical studies and a study protocol paper will be published to avoid selective outcome reporting.

### Ethics procedures

Considering that this study involves human subjects, specifically children who are considered a vulnerable group, it is important to obtain ethics clearance from the relevant ethics boards. This study has obtained ethics clearance from the Ethics Review Boards of Bielefeld University (number 2018–234) and the University of Dar es Salaam (number AB3/12(B)), Tanzania. During data assessment, only pre-assigned codes will appear on the questionnaires. Data will be stored on a password-secured computer accessible only to the study investigators. Personal data obtained during the research will be kept confidential and will not be disclosed to any other person without the participant’s permission or as required by the law. Behavioral intervention studies are minimum risk studies. In the event that there are any unexpected adverse effects, however, the researchers will document and report such occurrences to the trained psychologist on the research team. In case the problem is severe, the researcher will report the problem to the respective ethics bodies within one week. Questions about experiences may evoke upsetting memories in the event that the participant experienced similar events in his or her life. Participants who will experience any severe psychological distress during the course of the data collection will be provided with psychological support by a research team member. For participants who experience adverse or unexpected events, appropriate referrals and follow-up for specialized services and further management will be made on a case-by-case basis.

### Data analysis

Pre-assessment data will be used to provide information about the prevalence of maltreatment and violence in different settings as well as children’s mental health and well-being. Longitudinal analysis will be carried out based on the groups as randomized (intention to treat). We will use the last-observation-carried-forward approach; i.e., in drop-outs we assume no change from pre-assessment to follow-up. Results will be presented including appropriate effects sizes and with a measure of precision (95% confidence intervals). Our main analysis of the primary and secondary outcomes will be time × group interaction effects using repeated multivariate analysis of variance (MANOVA). In case of a noted cluster effect (intra-cluster correlation coefficient > 0.10) we will use multilevel analysis. Multivariate interaction effects and univariate interaction effects of each outcome variable will be tested first. Paired *t*-test analysis will examine the differences from the pre- to follow-up assessment in the intervention group while independent *t*-test will examine whether there is a difference between the control group and intervention group at the follow-up assessment. Effect size η^2^ ≥ 0.01, η^2^ ≥ 0.06 and η^2^ ≥ 0.14 will be considered to represent a small, moderate, and large effect, respectively [57]. For *t*-tests, effect size interpretation will be guided by the suggestion of Cohen where d ≥ 0.20, d ≥ 0.50, and d ≥ 0.80 will represent a small, medium, and large effect, respectively [57].

## Discussion

Violence against children by teachers at schools is a problem of global concern [7]. Previous research has shown that children are frequently exposed to violence by teachers in Tanzania [1, 5]. Students are frequently beaten and even experience severe forms of violence by teachers, such as being burnt [20]. The prevalence of violence against children in the Tanzanian school setting remains high in part due to the legality of violent discipline [48, 49] as well as the positive societal beliefs held by the community in general and by teachers in particular that violence is an effective and necessary way to control students’ behavior [6, 27]. Despite the high prevalence of violence against children, few school-based interventions that aim at reducing violence by teachers have been implemented in East Africa so far, and even fewer have been scientifically evaluated for their effectiveness [45]. In line with the United Nations’ Convention on the Rights of the Child [12] as well as the United Nation’s Sustainable Development Goal No. 16.2 [13], we argue that there is a strong need for conducting intervention studies that aim at reducing violent discipline by teachers in primary schools in Tanzania. Despite the existing reports on violence against children in schools in Tanzania [1, 5, 6, 15, 20], no such study has been carried out at the primary school level in Tanzania thus far.

Our study aims to implement and evaluate the* ICC-T* intervention approach. *ICC-T* aims to equip teachers with non-violent action alternatives and to encourage them to question their common beliefs and attitudes towards the effectiveness of violent disciplinary strategies in the classroom. *ICC-T* is an interactive, practical intervention in which teachers learn how they can implement non-violent disciplinary strategies in their daily work at school. Involving teachers in transforming and formulating their own training can help to promote engagement in this process. Reflections on teachers’ own experiences of violent discipline, discussions on the consequences of violence against children, and the intensive practice of effective non-violent disciplinary strategies can facilitate a change of attitudes towards violent discipline (see [1, 2, 6] for details). We therefore believe that the intervention can enable teachers to visualize the connection between violence and its negative consequences. This can convince teachers to implement alternative disciplinary approaches in schools. Furthermore,* ICC-T *has already been shown to be easily applicable in school settings in low-income countries [2].

In accordance with a similar trial currently being conducted in Uganda [2], our study will adopt a two-arm matched cluster randomized controlled trial design, with six schools allocated to the intervention group and six to the monitoring group. With our longitudinal and experimental design, we aim to test the feasibility and effectiveness of the *ICC-T* intervention approach for the first time at the primary school level in Tanzania using a scientifically rigorous design. The study will use a representative sample of students and teachers from public primary schools in Tanzania. Randomization of schools will control for most potential confounds, and the experimental design will allow a clear assignment of the effects to the intervention. Furthermore, our results can be generalized to similar school settings in Tanzania and the East-African region. The study follows a multi-information approach by collecting data from both teachers and students. The teachers’ self-reports will therefore be partly complemented by the students’ points of view. In addition, the assessment tools used in the study have a good theoretical basis and have proved reliable in measuring students’ exposure to and teachers’ use of and attitudes towards violence in schools and in examining mental health problems in East Africa [1, 5, 21, 33, 38, 54].

Following the successful evaluation of *ICC-T* at the secondary school level in Tanzania [1], we expect that it will be possible to change teachers’ attitudes and behavior in regard to violent discipline in the classroom. The successful implementation of *ICC-T* in public schools will contribute to the achievements of goal 16.2 of the United Nations Goals on Sustainable Development 2015–2030, which aim to end all forms of violence against children. It will also contribute to national efforts, such as the Tanzanian National Action Plan to End Violence Against Women and Children [58], that also aim to end all forms of violence against children in all settings in Tanzania.

Due to the longitudinal and experimental nature of this study, we are prepared to face a number of challenges which may result in limitations of the study. The potential challenges include some eligible participants refusing to participate in the study, as well as potential drop-outs at any stage of the project. Attrition among participating students and teachers may occur for a number of reasons, including possible transfers from one school to another as well as truancy and absenteeism. In respect to individual’s right to participation, the study team will ensure that participation in the study is entirely voluntary and any participant seeking to drop out from the study at any stage is free to do so. Though we will do our best to keep the attrition rate as low as possible, this may affect the potential findings. In addition, self-reported questionnaires are susceptible to possible bias of respondents and to social desirability. Furthermore, there are strong socio-cultural factors, attitudes, and beliefs that support the use of violence against children. The expected changes in attitudes and behavior can be considered only preliminary. In addition, the inclusion of relatively few schools limits the generalizability of the study results.

Despite these potential limitations, we believe that the study will contribute important new findings regarding the feasibility and effectiveness of *ICC-T* as one school-based preventative intervention approach to reduce violence by teachers. Furthermore, we presume that our study will contribute significantly to the global campaign to end violence against children [3, 4, 49] and therefore support the efforts made by the government of Tanzania to reduce violence against children in all settings. We therefore anticipate that, with successful evaluation of the *ICC-T* at the primary school level, the government, non-governmental organizations, policy makers, and other education stakeholders in Tanzania and other Sub-Saharan Africa countries will recognize the potential of school-based interventions in general and *ICC-T* in particular. It is hoped that this will lead relevant stakeholders to consider possibilities to scale up the intervention at the regional or national levels, integrating it in the regular teacher training programs.

## Trial status

The trial preparation phase is completed. Intervention pilot implementation took place in June 2019. Pre-assessment (control and intervention schools) was conducted from April (recruitment started on April 8th, 2019) to November 2019. Recruitment was completed by 30 November 2019. Interventions are planned in two phases: June and December 2019. The follow-up phase will start in Janary 2020 and end by May 2020 (study protocol number 1.4, 1^st^ July 2019).

## Supplementary information

**Additional file 1.** SPIRIT 2013 Checklist: Recommended items to address in a clinical trial protocol and related documents.

## Data Availability

The datasets generated and/or analyzed during the current study will be available from the corresponding author on request.

## Abbreviations

CTS: Conflict Tactics Scale
DE: Design effect
ICC: Interaction Competencies with Children
ICC-C: Interaction Competencies with Children for Caregivers
ICC-T: Interaction Competencies with Children for Teachers
MANOVA: Multivariate Analysis of Variance
SDQ: Strength and Difficulties Questionnaire
